# Using depth-enhanced diffuse correlation spectroscopy and near-infrared spectroscopy to isolate cerebral hemodynamics during transient hypotension

**DOI:** 10.1117/1.NPh.10.2.025013

**Published:** 2023-06-05

**Authors:** Leena N. Shoemaker, Daniel Milej, Jigneshkumar Mistry, Keith St. Lawrence

**Affiliations:** a Lawson Health Research Institute, Imaging Program, London, Ontario, Canada; b Western University, Department of Kinesiology, London, Ontario, Canada; c Western University, Department of Medical Biophysics, London, Ontario, Canada

**Keywords:** near-infrared spectroscopy, diffuse correlation spectroscopy, cerebral blood flow, hypotension

## Abstract

**Significance:**

Combining diffuse correlation spectroscopy (DCS) and near-infrared spectroscopy (NIRS) permits simultaneous monitoring of multiple cerebral hemodynamic parameters related to cerebral autoregulation; however, interpreting these optical measurements can be confounded by signal contamination from extracerebral tissue.

**Aim:**

We aimed to evaluate extracerebral signal contamination in NIRS/DCS data acquired during transient hypotension and assess suitable means of separating scalp and brain signals.

**Approach:**

A hybrid time-resolved NIRS/multidistance DCS system was used to simultaneously acquire cerebral oxygenation and blood flow data during transient orthostatic hypotension induced by rapid-onset lower body negative pressure (LBNP) in nine young, healthy adults. Changes in microvascular flow were verified against changes in middle cerebral artery velocity (MCAv) measured by transcranial Doppler ultrasound.

**Results:**

LBNP significantly decreased arterial blood pressure (−18%±14%), scalp blood flow (>30%), and scalp tissue oxygenation (all p≤0.04 versus baseline). However, implementing depth-sensitive techniques for both DCS and time-resolved NIRS indicated that LBNP did not significantly alter microvascular cerebral blood flow and oxygenation relative to their baseline values (all p≥0.14). In agreement, there was no significant reduction in MCAv (8%±16%; p=0.09).

**Conclusion:**

Transient hypotension caused significantly larger blood flow and oxygenation changes in the extracerebral tissue compared to the brain. We demonstrate the importance of accounting for extracerebral signal contamination within optical measures of cerebral hemodynamics during physiological paradigms designed to test cerebral autoregulation.

## 1 Introduction

The ability to monitor tissue oxygen saturation (${\mathrm{StO}}_{2}$) non-invasively by near-infrared spectroscopy (NIRS) has garnered considerable interest in clinical scenarios associated with a risk of brain damage, including certain surgical procedures and during intensive care.^1^ More recently, the combination of NIRS with diffuse correlation spectroscopy (DCS)^2^ offers the opportunity to simultaneously measure multiple cerebral hemodynamic and metabolic parameters, including cerebral blood flow (CBF), blood volume, and markers of oxygen metabolism.^3^^–^^7^ In addition to clinical bedside monitoring,^4^^,^^8^^–^^11^ both DCS and NIRS have the temporal resolution to measure transient changes in cerebral hemodynamics in response to various physiological paradigms designed to assess the mechanisms regulating CBF. These paradigms include hypercapnia to assess chemoregulation,^3^ head-up tilt,^12^ and thigh-cuff release^13^^,^^14^ to assess autoregulation related to vessel compliance and myogenic tone, and functional activation to assess neuronal control.^15^

Traditionally, non-invasive cerebral autoregulation has been assessed using transcranial Doppler ultrasound (TCD) to track changes in middle cerebral artery blood velocity (MCAv). While TCD is also non-invasive, it requires a constant and precise location of the ultrasound probe and cannot be used for long-term monitoring. Furthermore, since TCD measures blood velocity and not flow, it relies on the assumption that vascular tone remains stable throughout the assessment.^16^ In contrast, the combination of DCS and NIRS offers the ability to measure blood flow regulation at the microvascular level, and the combination of microvascular CBF and ${\mathrm{StO}}_{2}$ provides a means of assessing the potential impact of altered flow regulation on oxygen delivery and energy metabolism in the brain.

Despite the potential of these non-invasive optical techniques to study cerebrovascular regulation, interpreting NIRS and DCS data can be confounded by signal contamination from extracerebral tissue since the depth to the brain in the adult head varies between 1 and 2 cm.^17^^–^^19^ Consequently, over 80% of the signal at source-detector distance of <3  cm originates from hemodynamic changes in the scalp, easily overshadowing the cerebral component and resulting in substantial errors in ${\mathrm{StO}}_{2}$ and CBF measurements.^19^^,^^20^ Several approaches have been developed to reduce the effects of scalp contamination on NIRS and DCS, with the most common being to acquire data at multiple source-detector distances ($r_{\mathrm{SD}}$).^21^^–^^23^ More advanced technologies, i.e., time-resolved and frequency-domain methods, have proven effective for enhancing the depth sensitivity of NIRS.^24^^–^^27^ Time-resolved detection has also been applied to DCS^28^; however, this approach is challenging due to the poorer signal-to-noise ratio of current DCS technology.^29^ Consequently, multi-distance continuous-wave DCS remains the most commonly used approach in neuromonitoring applications. The substantially higher blood flow in the brain compared to the scalp gives DCS an inherent advantage in terms of depth sensitivity compared to NIRS.^30^

The importance of incorporating methods to separate cerebral and extracerebral signal contributions is particularly relevant to physiological stimuli considering many have systemic effects that likely alter scalp blood flow. For example, by collecting time-resolved NIRS (trNIRS) data at short and long source-detector distances ($r_{\mathrm{SD}}=1$ and 3 cm), Milej et al.^31^^,^^32^ demonstrated different vasoreactivity in scalp and brain in response to hypercapnia. If DCS and NIRS are to be used reliably in clinical applications involving bedside neuromonitoring, it is critical to understand the impact of extracerebral signal contributions on CBF and ${\mathrm{StO}}_{2}$ measurements during physiological perturbations, such as by changes in posture.

The purpose of this study was to investigate signal contamination from changes in scalp blood flow during transient hypotension.^13^ Measuring the CBF response to changes in blood pressure is a well-established method for characterizing cerebral autoregulation.^33^ Therefore, it is critical to evaluate the magnitude of extracerebral signal contamination in NIRS and DCS data and assess the suitability of technique-relevant methods for separating scalp and brain signals. For this study, a hybrid trNIRS/multidistance DCS system was used to acquire oxygenation and blood flow data during a rapid-onset lower body negative pressure (LBNP) challenge. This paradigm is a commonly used technique to mimic orthostatic stress (i.e., standing up) while the participant remains in a supine posture and is frequently used to assess cerebral autoregulation with TCD.^34^ Analysis of multidistance DCS data was based on the modified Beer-Lambert approach proposed by Baker et al.^35^ This approach requires applying pressure to the scalp to evaluate the sensitivity of DCS probes at different source-detector distances to scalp blood flow. In addition, a pneumatic tourniquet wrapped around the head was used to reduce scalp blood flow. The LBNP experiment was repeated with the tourniquet fully inflated to temporarily restrict scalp blood flow, thereby isolating the CBF response.^31^ Similarly, the influence of changes in scalp oxygenation on trNIRS measurements of ${\mathrm{StO}}_{2}$ and cerebral blood volume during LBNP was investigated. Time gates and moment analysis were applied to the trNIRS data to extract signals weighted to the superficial tissue (i.e., early-arriving photons) and brain (i.e., late-arriving photons). Finally, TCD was used to measure changes in MCAv in response to LBNP for comparison to the microvascular CBF responses measured by DCS.

## 2 Methods

### 2.1 Instrumentation

All data were collected using an in-house built trNIRS/DCS system.^3^^,^^36^ For the DCS module, light from a continuous laser with a long coherence length (>10  m) emitting at λ=852  nm (DL852-100-SO, CrystaLaser) was coupled into a single fiber (fiber core diameter 400  μm, NA=0.22, Loptek, Germany). On the detection side, reflected light was collected by one single-mode fiber at a source-detector separation ($r_{\mathrm{SD}}$) of 1 cm and three fibers at $r_{\mathrm{SD}}=2.5\;\mathrm{cm}$ (core diameter 8  μm, NA=0.12, Loptek, Berlin, Germany). Detection fibers were coupled to a four-channel single-photon counting module (SPCMAQR-15-FC, Excelitas Technologies, Canada). Each counting module generated TTL pulses sent to an edge-detecting photon counter on a PCIe6612 counter/timer data acquisition board (National Instrument).^37^ Photon counts were recorded and processed using in-house developed software written in LabVIEW (2019, National Instruments) and MATLAB (2017, MathWorks). Intensity autocorrelation curves were generated for 50 delay times (τ) ranging from 1μ to 1 ms.

The trNIRS module included two pulsed laser heads emitting at λ=760 and 830 nm, controlled by a Sepia II laser driver operating at 80 MHz (PicoQuant, Germany). The laser heads were coupled to multimode bifurcated fiber (core diameter 400  μm, NA = 0.39, Loptek, Germany) to deliver the light to the scalp. A detection fiber bundle (3.6 mm, NA = 0.55, Loptek, Germany) collected diffusively reflected light from the scalp at $r_{\mathrm{SD}}=3\;\mathrm{cm}$ and was coupled to a hybrid photomultiplier tube (PMA Hybrid 50, PicoQuant, Germany).^38^ A time-correlated single-photon counting module (HydraHarp 400, PicoQuant, Germany) was used to record photon arrival times and generate a time-of-flight distribution of diffusely reflected photons (DTOF).^32^^,^^39^ To acquire trNIRS/DCS data simultaneously, a short-pass interference filter was placed between collimating lenses in front of trNIRS detector (Spec 3551, 836.5 nm, diameter 25 mm, Alluxa, Santa Rosa, California, United States).^3^ At the end of every study, the instrument response function (IRF) was measured using a custom-built light-tight box that connected the emission fiber to a detection probe with a separation of 10 cm.

### 2.2 Experimental Procedure

All procedures were approved by the Health Sciences Research Ethics Board at Western University (Grant No. 120391) and Lawson Health Research Institute (Grant No. 107985), adhering to the guidelines of the Tri-Council Policy Statement for research involving humans. Participants provided written informed consent following verbal and written explanations of the experimental procedures.

**Fig. 1:**
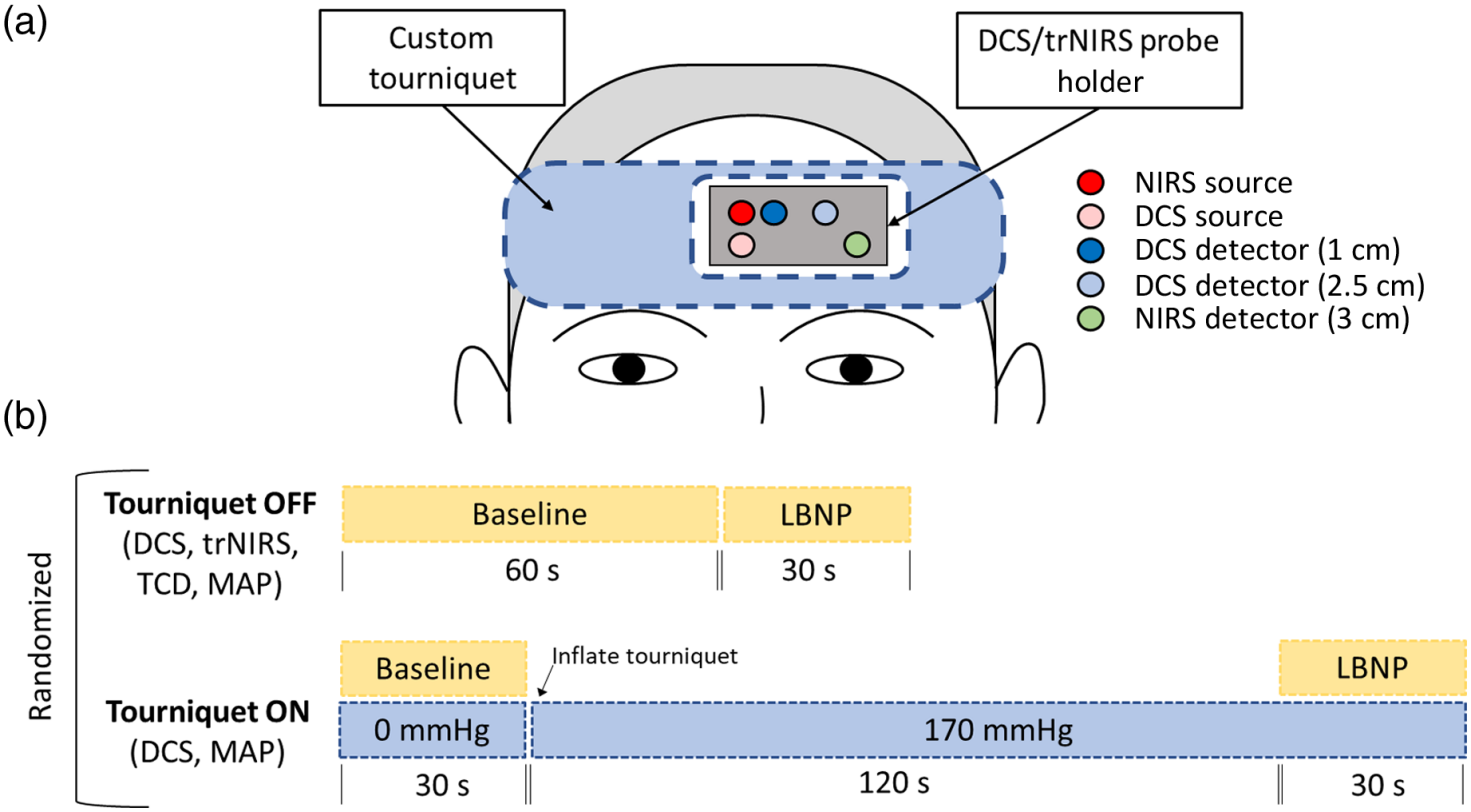
(a) Illustration of optical probes placement and (b) experimental paradigms used in the study. Light sources and detectors on the probe holder are color-coded. Red, NIRS source; pink, DCS source; dark blue, DCS detector ($r_{\mathrm{SD}}=1\;\mathrm{cm}$); light blue, DCS detector ($r_{\mathrm{SD}}=2.5\;\mathrm{cm}$); green, trNIRS detector ($r_{\mathrm{SD}}=3\;\mathrm{cm}$).

Ten healthy participants (5 women, 26±4 years, 177±6  cm, 75±15  kg) with no history of any neurological or psychiatric disorders were recruited. All participants identified with the sex that was assigned to them at birth. Participants completed two protocols, one with the tourniquet [Fig. 1(a)] off and the other with the tourniquet on [Fig. 1(b)]. To create a rapid but brief (15 s) bout of orthostatic stress, participants were sealed from the waist down in an LBNP chamber. The cerebral hemodynamic responses were assessed for a short (15 s) pulse of rapid-onset LBNP with suction levels of −80  mmHg. For this protocol, cerebrovascular hemodynamics were assessed with DCS, trNIRS, and TCD. All three were acquired continuously throughout the LBNP protocol at a sampling rate of 3.33 Hz for trNIRS/DCS and 3.33 Hz for TCD.

The DCS and trNIRS probes were secured to the forehead using a custom 3D-printed holder made of flexible resin (Flexible 80A, Formlabs, Somerville, Massachusetts, United States). Confounding effects related to placing probes close to the sagittal sinus were considered; however, Liu et al.^40^ showed that NIRS measurements are primarily sensitive to oxygenation in the microvasculature as almost all light that interrogates larger vessels, such as the sagittal sinus, is absorbed. Placing the probes on the midline may have resulted in a greater loss of light, but the signals from both trNIRS and DCS were found to be adequate for this study. A finger photoplethysmography was used to provide continuous arterial blood pressure (ABP) recordings during LBNP (Finometer, Finapres Medical Systems, Enschede, The Netherlands). Mean ABP (MAP) was calculated using the algebraic mean for each cardiac cycle. The finger unit was calibrated against three manual sphygmomanometric brachial artery measures. Expired gas was sampled at the mouth to measure partial pressure end-tidal ${\mathrm{CO}}_{2}$ ($P_{\mathrm{ET}}{\mathrm{CO}}_{2}$; ML206 analyser). Finally, participants were instrumented with TCD (Neurovision TOC2M, Multigon Industries, Elmsford, New York, United States) for measures of right MCAv (M1 segment). The ultrasound probe was held in place with a headband device.

As a means of assessing scalp blood flow contributions to the DCS signal, the LBNP experiment was repeated after inflating a 10-cm wide tourniquet wrapped around the head, just above the supraorbital ridge.^31^ The tourniquet was designed to impede blood flow to the scalp by inflating two bladders positioned over the temples. The tourniquet included an opening for the optical probe holder (9  cm×5  cm) that was large enough to ensure the tourniquet did not press down on the holder when inflated [Fig. 1(a)]. DCS data were acquired continuously while inflating the tourniquet to 170 or 50 mmHg above systolic blood pressure for 120 s. The tourniquet remained at the higher pressure while the LBNP protocol was repeated [Fig. 1(b)].

### 2.3 Data Analysis

#### 2.3.1 trNIRS

To determine baseline optical properties for each subject, a mean DTOF was generated from the first minute of baseline recordings (i.e., prior to LBNP) for each wavelength. Each DTOF was fit with the solution to the diffusion equation for a semi-infinite homogeneous medium convolved with the measured IRF (fminsearch, MATLAB, MathWorks Inc., Natick, Massachusetts, United States). The fitting parameters were the baseline absorption coefficient ($\mu_{a0}$), reduced scattering coefficient ($\mu_{s0}^{\prime }$) and an amplitude factor that accounts for laser power, detection gain, and coupling efficiency. The fitting range was set to 10% of the peak value of a DTOF on the leading edge and 1% on the falling edge.

Next, each DTOF in a time series recorded at 760 and 830 nm was analyzed to calculate the first three statistical moments: the number of photons (N), the mean time of flight (⟨t⟩), and the variance (V). The moments were calculated by setting the lower and upper integration limits based on arrival times corresponding to 1% of the peak of the DTOF.^41^^,^^42^ The change in each moment relative to its initial baseline value was calculated to generate three time series (i.e., ΔN, Δ⟨t⟩, and ΔV) for the two wavelengths individually. Time courses for each statistical moment were converted into absorption changes ($\Delta \mu_{a}$) using sensitivity analysis as described in detail elsewhere.^32^^,^^43^ The sensitivity factors were generated using subject-specific baseline optical properties obtained through the fitting routine described above (mean $\mu_{a0}=0.12\pm 0.03\;{\mathrm{cm}}^{-1}$ and $\mu_{s0}^{\prime }=9.2\pm 1.2\;{\mathrm{cm}}^{-1}$ at 760 nm; mean $\mu_{a0}=0.13\pm 0.03\;{\mathrm{cm}}^{-1}$ and $\mu_{s0}^{\prime }=8.9\pm 1.2\;{\mathrm{cm}}^{-1}$ at 830 nm). The $\Delta \mu_{a}$ time courses recorded at 760 and 830 nm were converted to changes in concentration of oxyhemoglobin ($\Delta C_{\mathrm{HbO}}$) and deoxyhemoglobin ($\Delta C_{\mathrm{Hb}}$) using their respective molar extinction coefficients.^44^ $\Delta C_{\mathrm{HbO}}$ and $\Delta C_{\mathrm{Hb}}$ time courses were used to determine the corresponding change in tissue oxygen saturation ($\Delta {\mathrm{StO}}_{2}$) using the standard definition of ${\mathrm{StO}}_{2}$: ${\mathrm{StO}}_{2}=C_{\text{HbO}}/(C_{\mathrm{HbO}}+C_{\mathrm{Hb}})$. The sum of $\Delta C_{\mathrm{HbO}}$ and $\Delta C_{\mathrm{Hb}}$ was also used to determine changes in the total hemoglobin concentration (ΔtHb), which reflects changes in blood volume, assuming no change in hemoglobin concentration.

In addition to moment analysis, two gates, one early and one late, were extracted from each DTOF to generate time-varying changes in attenuation (ΔA) with sensitivity to the scalp and cerebral oxygenation, respectively.^32^ Both gates had a width of 250 ps.^45^ The start of the early gate was positioned at the rising edge of the DTOF when the signal intensity reached 1% of the peak value, whereas the end of the late gate was located on the opposite DTOF edge where the intensity dropped to 1%. Analogous to moment analysis, ΔA time series for the two gates measured at the two wavelengths were converted to $\Delta C_{\mathrm{HbO}}$, $\Delta C_{\mathrm{Hb}}$ using corresponding sensitivity factors.^32^ Similar to the statistical moment analysis described above, time courses of $\Delta C_{\mathrm{HbO}}$ and $\Delta C_{\mathrm{Hb}}$ were used to calculate $\Delta {\mathrm{StO}}_{2}$ and ΔtHb. All time courses were smoothed with a 0.9-s (three data points) moving average with a zero-phase digital filter (filtfilt, MATLAB, MathWorks Inc., Natick, Massachusetts, United States).

#### 2.3.2 DCS—semi-infinite model

Normalized intensity autocorrelations functions were converted to electric field autocorrelation data using the Siegert relation.^46^ Each autocorrelation function was subsequently fit with the solution to the diffusion approximation for a semi-infinite homogenous medium to estimate the blood flow index (BFi), assuming tissue perfusion is modeled as a pseudo-Brownian motion.^2^ The fitting incorporated each subject’s $\mu_{a0}$ and $\mu_{s0}^{\prime }$ values measured by trNIRS. Fitting was performed across all correlation times from 1  μs to 1 ms. The resulting BFi time courses were smoothed with the same filter (zero-phase digital filtering, filtfilt, MATLAB, 2016b, MathWorks, Natick, Massachusetts, United States) as the trNIRS data.

#### 2.3.3 DCS—multilayered model

Depth-enhanced DCS analysis was based on the probe pressure modulation algorithm for CBF monitoring described by Baker et al.^35^ Based on a two-layer modified Beer-Lambert approach, changes in CBF during LBNP were extracted from the multidistance DCS data using

$$\Delta F_{C}(\tau ,t)=\frac{1}{\delta F_{C}(\tau )}[\Delta {\mathrm{OD}}_{L}(\tau ,t)-\frac{\Delta {{\mathrm{OD}}_{L}}^{P}(\tau )}{\Delta {{\mathrm{OD}}_{S}}^{P}(\tau )}\Delta {\mathrm{OD}}_{S}(\tau ,t)],$$ (1)

where, $\delta F_{C}$, represents the sensitivity to the brain layer for DCS data recorded at $r_{\mathrm{SD}}=2.5\;\mathrm{cm}$. $\Delta {\mathrm{OD}}_{S}(t)$ and $\Delta {\mathrm{OD}}_{L}(t)$ are the changes in the DCS optical density measured at the short (1 cm) and long (2.5 cm) distances, respectively. $\Delta {{\mathrm{OD}}_{S}}^{P}$ and $\Delta {{\mathrm{OD}}_{L}}^{P}$ are the changes in optical density measured in response to a change in scalp blood flow. In these experiments, $\Delta {{\mathrm{OD}}_{S}}^{P}$ and $\Delta {{\mathrm{OD}}_{L}}^{P}$ were obtained from inflating the head tourniquet. A change in optical density at a given source-detector distance is defined by

$$\Delta {\mathrm{OD}}_{r_{\mathrm{SD}}}(\tau ,t)=-\log[\frac{g_{2}(\tau ,r_{\mathrm{SD}})-1}{g_{2}^{0}(\tau ,r_{\mathrm{SD}})-1}],$$ (2)

where, $g_{2}(\tau ,r_{\mathrm{SD}})$ is the measured intensity autocorrelation function.

Baseline blood flow indices for scalp and brain ($F_{S,0}$ and $F_{C,0}$, respectively) were estimated using a three-layered DCS model that represents blood flow in scalp, skull and brain.^23^ The model was applied to intensity autocorrelation data acquired at $r_{\mathrm{SD}}=2.5\;\mathrm{cm}$ before and after inflating the tourniquet. Analysis was performed assuming $F_{C,0}$ did not change between the two conditions and skull blood flow was set to 1% of $F_{S,0}$ since bone blood flow is expected to be low.^47^ $\mu_{s0}^{\prime }$ was assumed to be the same in all three layers and set to the value measured by trNIRS for each subject. Similarly, $\mu_{a0}$ for the brain and scalp was set to subject-specific values from trNIRS, and $\mu_{a}$ for the skull was set equal to 60% of $\mu_{a0}$ based on Strangman et al.^48^

The thicknesses of the scalp and skull layers were set to average values from a previous study: 0.65 cm for scalp and 0.55 cm for the skull.^36^ The final step was to estimate $\delta F_{C}$, which was obtained from the three-layered model for a 1% change in CBF and using subject-specific values of $F_{S,0}$ and $F_{C,0}$. For the data obtained for a fully inflated (170 mmHg) head tourniquet, the change in optical density at $r_{\mathrm{SD}}=2.5\;\mathrm{cm}$ measured for each subject was scaled by subject-specific $F_{C,0}$ and $\delta F_{C}$. Since the distance to the brain ($d_{SC}$) was not measured for each subject, the analysis described above was performed for $d_{SC}$ values from 1 to 1.4 cm, assuming a fixed ratio of the scalp to skull thicknesses of 1.2.

#### 2.3.4 Numerical simulations

For comparison to the experiment data, the three-layered model was used to generate simulated data at $r_{\mathrm{SD}}=1$ and 2.5 cm over a range of $d_{SC}$ values from 0.9 to 1.4 cm and using average values of $\mu_{a0}=0.13\;{\mathrm{cm}}^{-1}$ and $\mu_{{s0}^{\prime }}=9\;{\mathrm{cm}}^{-1}$. Simulated data were generated for a reduction in scalp blood flow of 85% to reflect the effect of the tourniquet and for comparison an 85% reduction in CBF. Data at the two source-detector distances were analyzed individually using the semi-infinite model to predict the expected change in BFi.

### 2.4 Statistical Analysis

All data are presented as mean ± standard deviation unless otherwise noted. Statistical significance was defined as p<0.05. Paired t-tests were used to assess the effect of LBNP on each variable (i.e., the last 5 s of LBNP compared to the 10-s baseline). For DCS variables, an additional paired t-test was used to assess the difference between CBF calculations derived from the multilayer model with and without the tourniquet inflation. A two-way analysis of variance (ANOVA) of time (two levels: baseline, nadir) and $r_{\mathrm{SD}}$ (two levels: 1 cm, 2.5 cm) evaluated the BFi response to LBNP between long and short $r_{\mathrm{SD}}$. For trNIRS, paired t-tests were used to evaluate differences between variance and both early or late gates and the early gate compared to the number of photons. Changes in MCAv were assessed using one-way repeated measures ANOVA with a fixed factor of time (10 levels: 3 s bins across 30 s) for LBNP trials at −80  mmHg.

## 3 Results

**Fig. 2:**
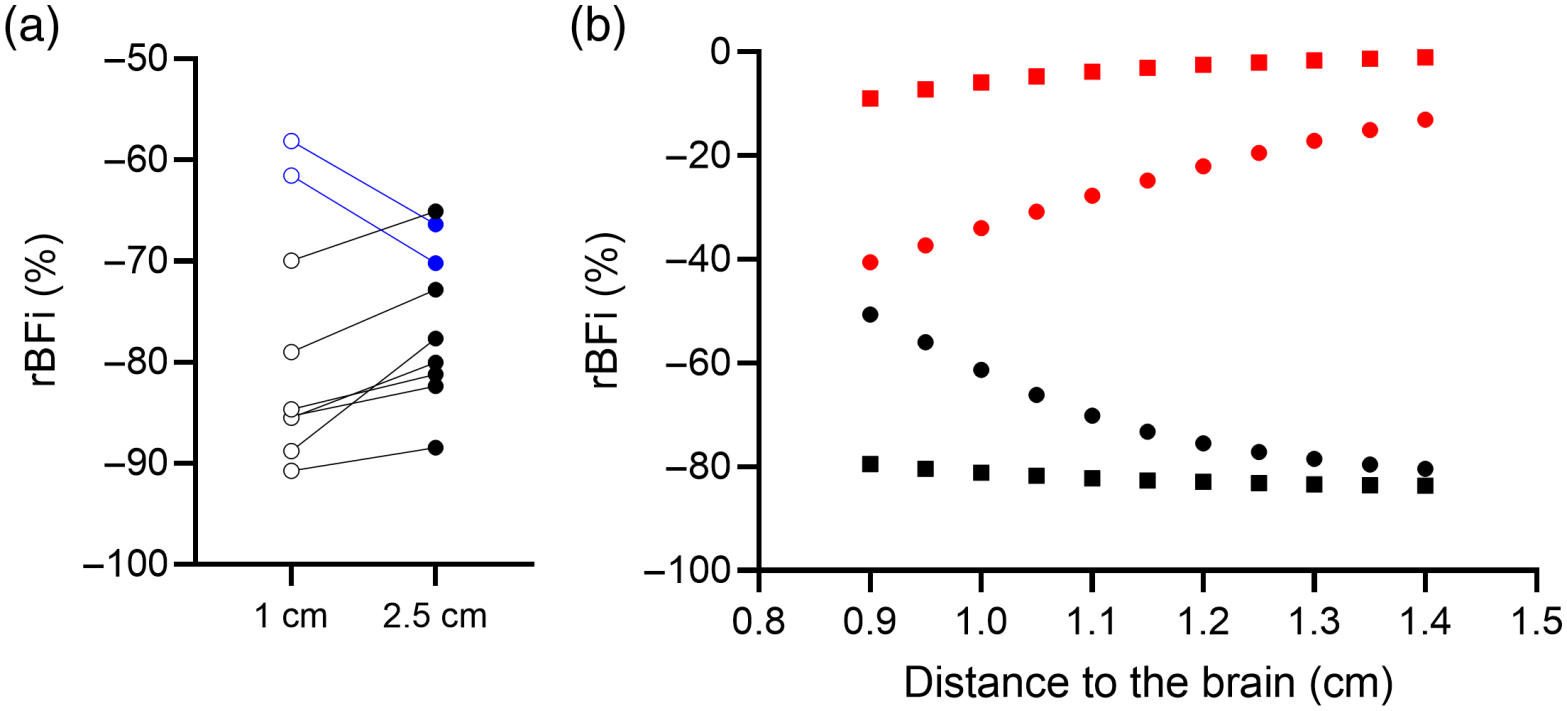
(a) Measured change in rBFi for each subject in response to inflating the tourniquet to 170 mmHg. (b) Predicted change in rBFi at two source-detectors distances ($r_{\mathrm{SD}}=1$, squares and 2.5 cm, circles) as a function of distance to the brain. Results are presented for an 85% reduction in scalp blood flow (black lines) and an 85% reduction in CBF (red lines).

Of the 10 participants, 1 was omitted due to large intensity fluctuations (>50% of the $g_{2}$ curve mean) in the autocorrelation curves and low photon count (<50  k) in the DTOFs, which were attributed to poor skin-to-probe contact. All participants tolerated the LBNP and tourniquet inflation. Figure 2(a) illustrates relative BFi (rBFi) changes measured at the two source-detector distances in response to inflating the head tourniquet to 170 mmHg. The rBFi reduction was greater at $r_{\mathrm{SD}}=1\;\mathrm{cm}$ than 2.5 cm for 7 out of 9 participants (all p<0.01), with an average difference of 5%±3% (n=7). For the remaining two participants, the measured rBFi reductions at $r_{\mathrm{SD}}=2.5\;\mathrm{cm}$ were within the range found for the other participants. The smaller responses measured at $r_{\mathrm{SD}}=1\;\mathrm{cm}$ may have been due to unequal pressure around the probes when the tourniquet was inflated. Due to this unexpected experimental issue, these two data sets were excluded from the calculation of $\Delta F_{C}$ using Eq. (1).

The numerical simulations [Fig. 2(b)] predicted a BFi decrease of 77% at $r_{\mathrm{SD}}=2.5\;\mathrm{cm}$ and 83% at $r_{\mathrm{SD}}=1\;\mathrm{cm}$ when the distance to the brain is 1.2 cm, which is the average distance on the forehead measured previously by magnetic resonance imaging (MRI).^36^ This prediction in good agreement with the experimental results. Of note, a change in CBF at the same distance is predicted to cause approximately a 20% change in the BFi measured at $r_{\mathrm{SD}}=2.5\;\mathrm{cm}$ and only 1.5% at $r_{\mathrm{SD}}=1\;\mathrm{cm}$.

**Fig. 3:**
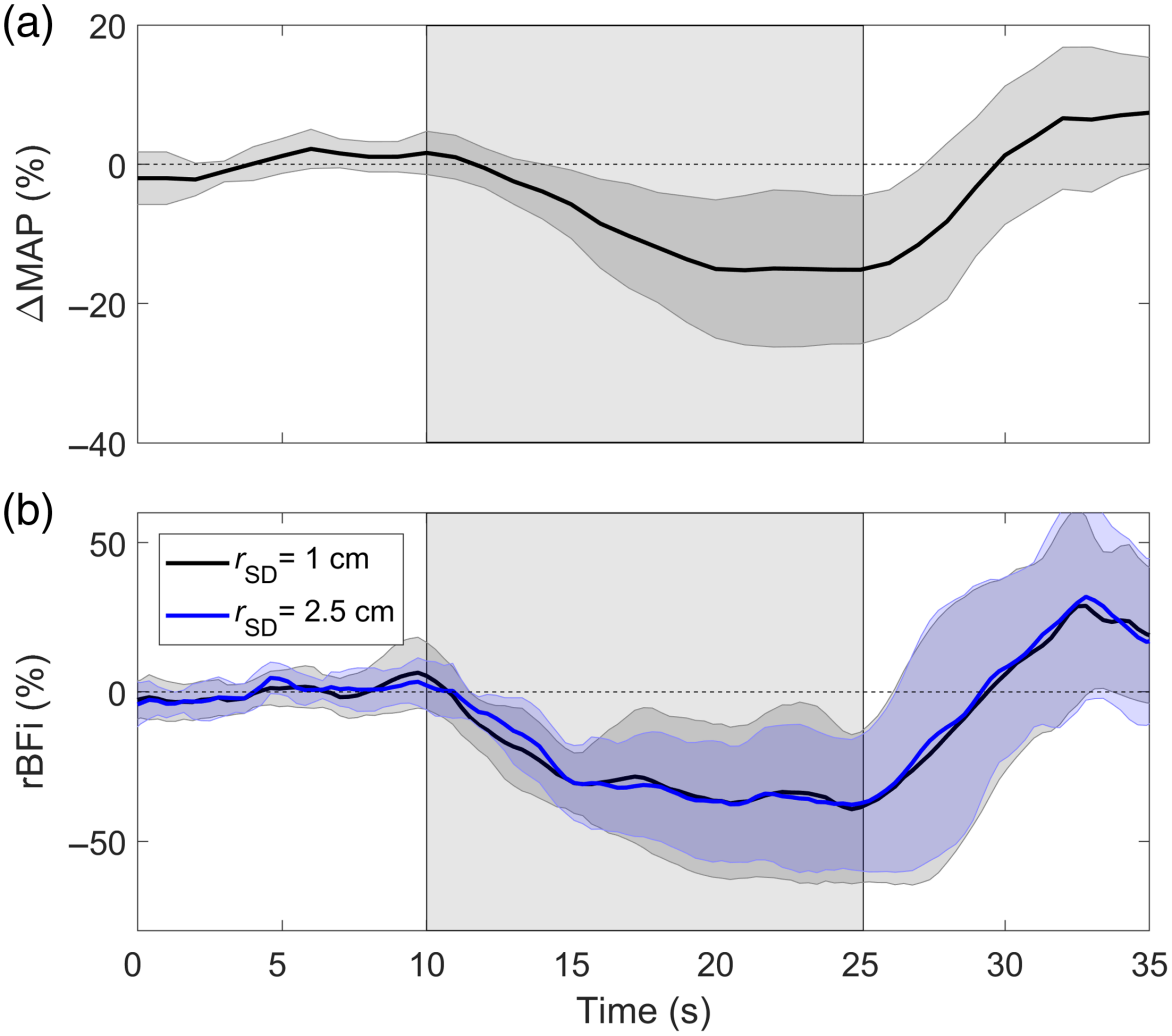
Average changes in (a) MAP and (b) rBFi plotted as a function of time during LBNP, which is indicated by the grey region between 10 and 25 s. DCS data were recorded at $r_{\mathrm{SD}}=1$ and 2.5 cm without inflating the head tourniquet. Shadowing surrounding each time course represents the standard deviation across subjects.

LBNP of −80  mmHg produced a significant decrease in MAP of −17±11  mmHg [−24%±16%; p<0.01; Fig. 3(a)]. The average change in MAP during LBNP after tourniquet inflation was not statistically different (−12±7  mmHg; p=0.12). End-tidal ${\mathrm{CO}}_{2}$ decreased slightly, albeit not significant during LBNP (37±4 to 33±8  mmHg, p=0.10). Corresponding time-varying changes in rBFi recorded at the two source-detector distances are presented in Fig. 3(b). Both distances showed a significant reduction in rBFi (p<0.01 versus baseline). The decrease calculated for the last 5 s of LBNP was −36%±25% for $r_{\mathrm{SD}}=1\;\mathrm{cm}$ and −36%±20% for $r_{\mathrm{SD}}=2.5\;\mathrm{cm}$. No statistical difference was observed in the responses obtained at the two source-detector separations ($\text{time}\times r_{\mathrm{SD}}$ interaction and the main effect of $r_{\mathrm{SD}}$: p=0.97).

**Fig. 4:**
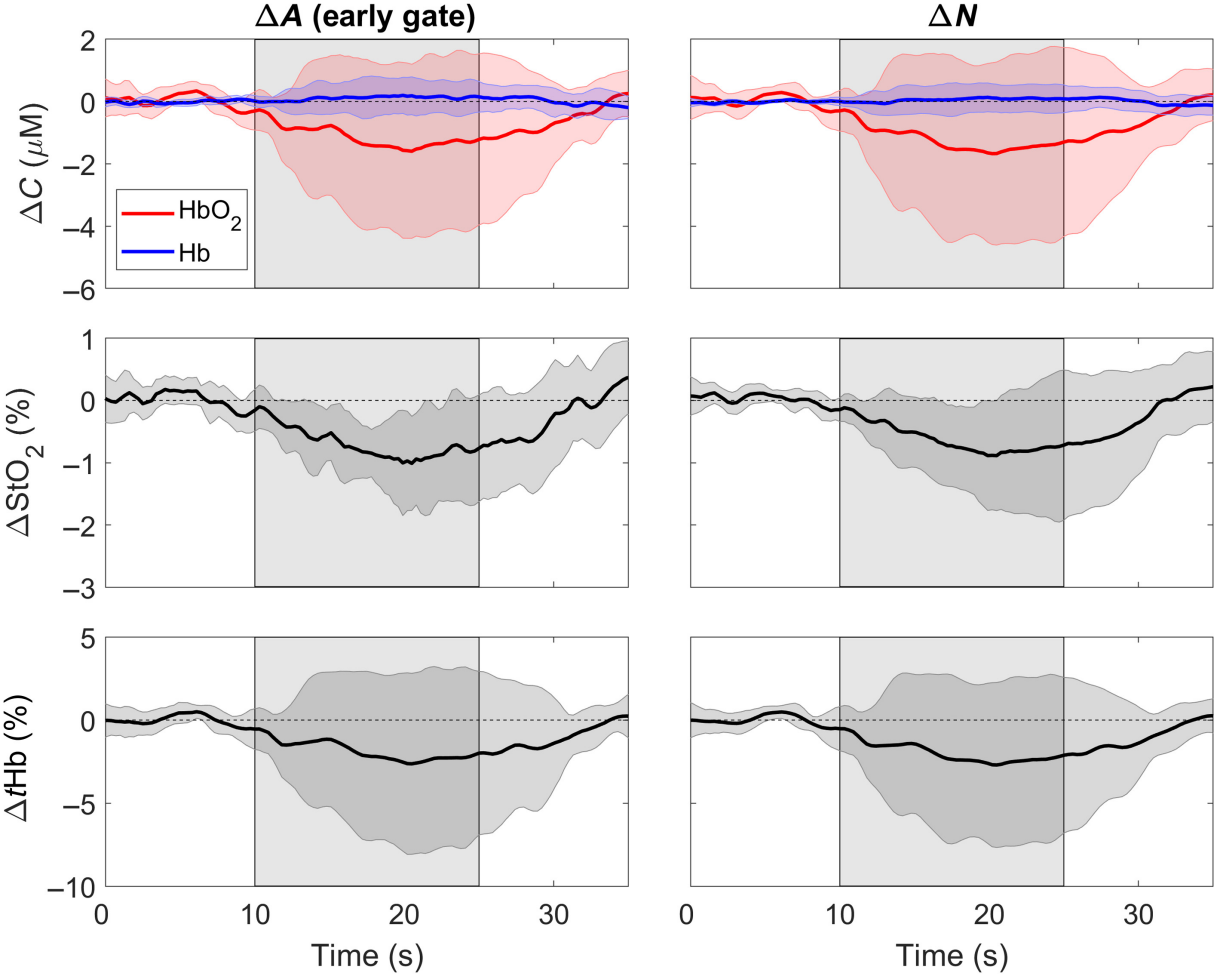
Average $\Delta C_{\mathrm{HbO}}$, $\Delta C_{\mathrm{Hb}}$ $\Delta {\mathrm{StO}}_{2}$, and ΔtHb responses to LBNP (between 10 and 25 s). Time courses are presented for the change in attenuation measured in an early time gate and from the total number of photons (ΔN). All time courses were averaged across nine subjects. Shadowing represents the standard deviation across subjects.

Time-varying changes in the NIRS parameters (i.e., $\Delta C_{\mathrm{HbO}}$, $\Delta C_{\mathrm{Hb}}$, $\Delta {\mathrm{StO}}_{2}$, and ΔtHb) during the LBNP challenge are presented in Fig. 4. These time courses were generated from the metrics with the poorest depth sensitivity, namely ΔA from the early gate and ΔN. For both measurements, decreases in ${\mathrm{StO}}_{2}$ during LBNP were statistically significant (both p≤0.04 versus baseline). No significant difference was found between $\Delta {\mathrm{StO}}_{2}$ obtained for the early gate and ΔN (p=0.62). LBNP did not drive a significant change in $C_{\mathrm{HbO}}$, $C_{\mathrm{Hb}}$ or ΔtHb for ΔA or ΔN (all p≥0.16 versus baseline).

**Fig. 5:**
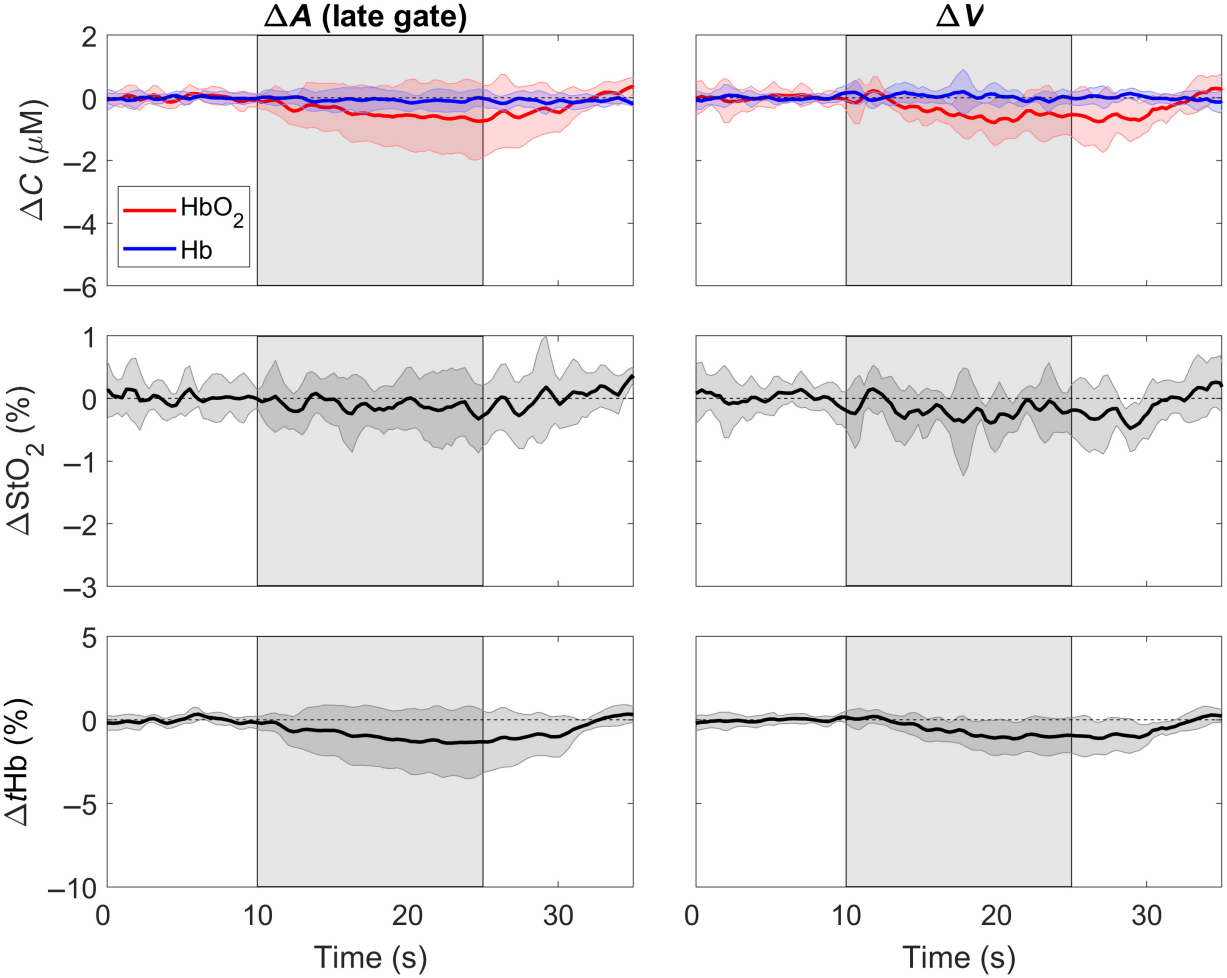
Average $\Delta C_{\mathrm{HbO}}$, $\Delta C_{\mathrm{Hb}}$, $\Delta {\mathrm{StO}}_{2}$, and ΔtHb responses to LBNP (between 10 and 25 s). Time courses are presented for metrics with greater depth sensitivity (i.e., ΔA from the late gate and the change in variance, ΔV). All time courses were averaged across nine subjects. Shadowing represents the standard deviation across subjects.

ΔCHbO, ΔCHb, $\Delta {\mathrm{StO}}_{2}$, and ΔtHb time courses derived from the two metrics with the highest depth sensitivity (i.e., ΔA from a late gate and ΔV) are presented in Fig. 5. No significant change occurred during LBNP for any variable obtained by the late gate and ΔV analyses (all p≥0.17). However, changes in $\Delta {\mathrm{StO}}_{2}$ obtained from the variance and late-gate were significantly smaller than the corresponding $\Delta {\mathrm{StO}}_{2}$ changes obtained from the early-gate analysis (p≤0.02).

**Fig. 6:**
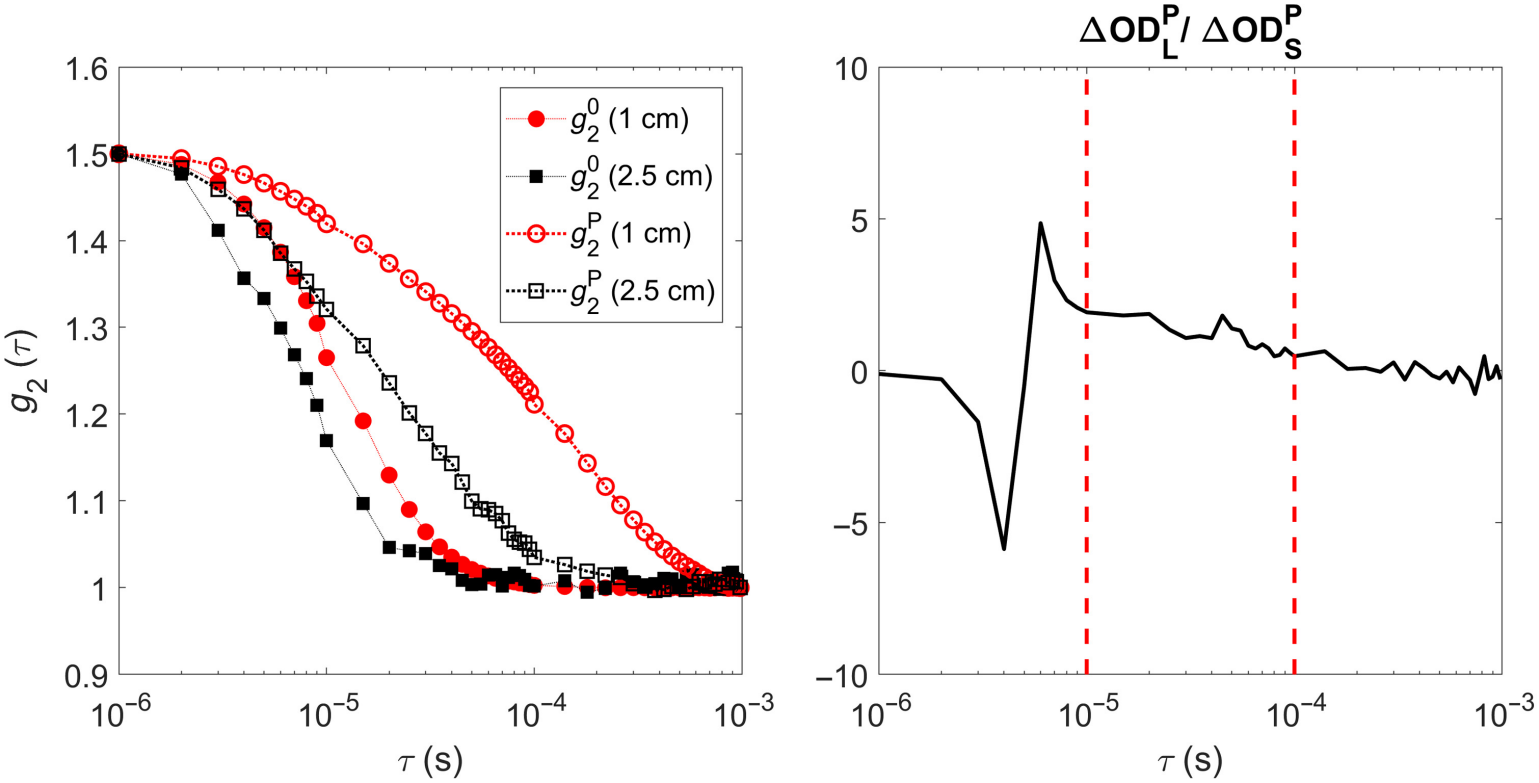
Representative autocorrelation curves (one subject) for the two source-detector separations (left) measured at baseline (superscript “0”) and after inflating the tourniquet (superscript “P”). Corresponding $\Delta {{\mathrm{OD}}_{L}}^{P}/\Delta {{\mathrm{OD}}_{S}}^{P}$ ratio as a function of correlation time is shown on the right. Dashed red lines represent a range of delay times τ selected to calculate the average $\Delta {{\mathrm{OD}}_{L}}^{P}/\Delta {{\mathrm{OD}}_{S}}^{P}$ ratio.

Figure 6 presents representative autocorrelation curves obtained at the baseline ($g_{2}^{0}$) and increased tourniquet pressure ($g_{2}^{P}$) measured at the two source-detector separations and the corresponding ratio of $\Delta {{\mathrm{OD}}_{L}}^{P}/\Delta {{\mathrm{OD}}_{S}}^{P}$. For each subject, $\Delta {{\mathrm{OD}}_{L}}^{P}/\Delta {{\mathrm{OD}}_{S}}^{P}$ was calculated for τ values from 10 to 100  μs, as this range avoided instabilities evident at shorter and longer delay times.^35^ Table 1 provides subject-specific baseline optical properties obtained with trNIRS, $\Delta {{\mathrm{OD}}_{L}}^{P}/\Delta {{\mathrm{OD}}_{S}}^{P}$ measurements, and estimates of $F_{S,0}$, $F_{C,0}$ and $\delta F_{C}$ from the three-layered DCS model. Note, $\Delta {{\mathrm{OD}}_{L}}^{P}/\Delta {{\mathrm{OD}}_{S}}^{P}$ values are not presented for the two subjects with the unexpected tourniquet response at the short $r_{\mathrm{SD}}$.

**Table 1:** Subject-specific estimates of baseline optical properties measured by trNIRS, $\Delta {{\mathrm{OD}}_{L}}^{P}/\Delta {{\mathrm{OD}}_{S}}^{P}$, baseline blood flows ($F_{S,0}$, $F_{C,0}$), and the cerebral sensitivity factor obtained from the three-layered DCS model.

Subject no.	Sex	$\mu_{a0}$, $\mu_{s0}^{\prime }$ (${\mathrm{cm}}^{-1}$)	$\Delta {{\mathrm{OD}}_{L}}^{P}/\Delta {{\mathrm{OD}}_{S}}^{P}$	$F_{S,0}$ ($10^{-8}{\cdot \mathrm{cm}}^{2}/s$)	$F_{C,0}$ ($10^{-8}{\cdot \mathrm{cm}}^{2}/s$)	$\delta F_{C}$ ($10^{8}\cdot s/{\mathrm{cm}}^{2}$)
1	F	0.14, 9.6	—	0.42	1.29	2.81
2	F	0.12, 8.7	0.77	0.75	2.25	2.01
3	M	0.08, 10.4	0.68	0.18	0.94	3.22
4	F	0.17, 6.6	1.10	0.75	1.66	2.51
5	M	0.12, 8.4	0.82	0.28	1.42	2.56
6	F	0.12, 8.5	0.93	0.41	2.25	1.91
7	F	0.11, 9.7	1.04	0.51	1.70	2.37
8	M	0.19, 10.9	—	0.25	3.92	1.18
9	M	0.12, 7.8	0.65	0.61	2.33	1.91
**Average ± SD**		**0.13 ± 0.03, 9.0 ± 1.3**	**0.86 ± 0.17**	**0.46 ± 0.21**	**1.97 ± 0.87**	**2.27 ± 0.60**

**Fig. 7:**
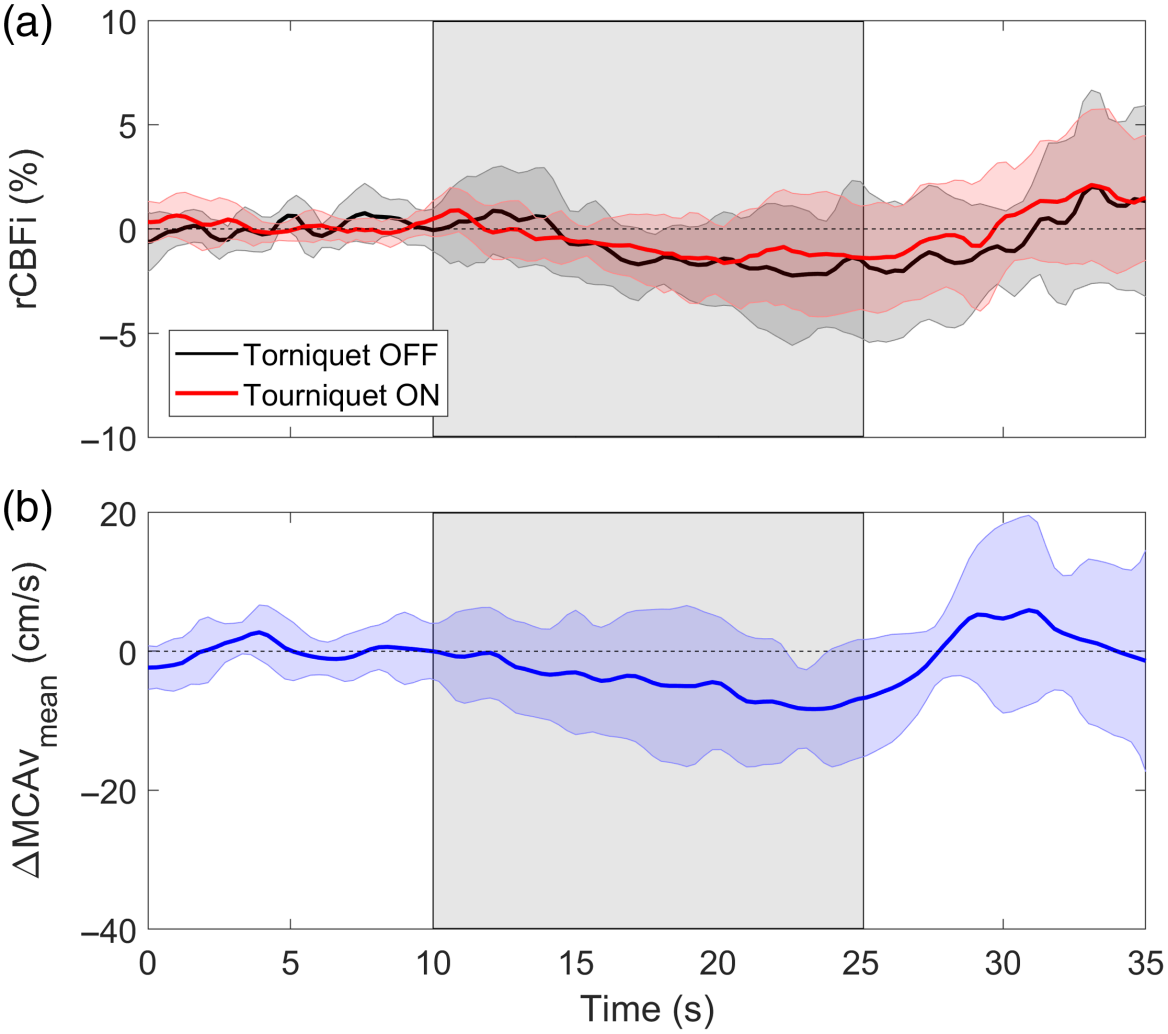
(a) Average relative CBF (rCBF) response to the LBNP challenge between 10 to 25 s. Time courses are shown for DCS data acquired with and without the tourniquet inflated. (b) Mean MCAv (${\mathrm{MCAv}}_{\text{mean}}$) during LBNP. TCD data were acquired simultaneous to DCS (the tourniquet off condition). Shadowing represents the standard deviation across seven subjects.

Figure 7(a) presents average time courses of the microvascular CBF response to LBNP across the seven subjects. Time courses are shown for DCS data acquired with or without the tourniquet inflated. For each subject, the CBF response derived using Eq. (1) was scaled by a subject-specific $F_{C,0}$, from the three-layered model. The reconstructed changes in CBF during LBNP [Fig. 7(a)] were significantly smaller than the BFi values obtained using the semi-infinite model (Fig. 3; p<0.01) and did not show a significant change during LBNP (both p≥0.13 versus baseline). Further, the reconstructed blood flow changes (−1.9%±2.8%) and the blood flow response measured after the tourniquet was inflated (−1.3%±2.6%) were not significantly different from each other (p=0.75). For comparison, the average MCAv response to LBNP is shown in Fig. 7(b). The MCAv response to LBNP was not significant across time. At the nadir, the average reduction was 8%±16% (one-way ANOVA: p=0.09).

## 4 Discussion

This study utilized a hybrid trNIRS/multidistance DCS system to assess the magnitude of extracerebral signal contamination in NIRS and DCS data acquired during transient hypotension (induced by LBNP) and the suitability of technique-relevant methods for separating scalp and brain measurements. The motivation was to improve the confidence when applying these non-invasive optical technologies to applications in critical-care settings, given the clinical interest in using flow and metabolic markers to assess brain health and prevent secondary brain injury. The primary outcome was demonstrating that transient hypotension caused significantly larger blood flow and oxygenation changes in the extracerebral tissue compared to the brain. Further, depth-enhanced methods proved effective at removing this signal contamination.

The impact of extracerebral signal contamination on DCS blood flow measurements was evaluated by acquiring data at two source-detector distances. A large (i.e., >30%), statistically significant decrease in blood flow was detected when data acquired at the two distances were analyzed separately using the semi-infinite model, which neglects the layered structure of the head. There was no significant difference in the magnitude of the blood flow responses measured at the two distances; moreover, the two time courses were extremely similar, as reflected by the non-significant $\text{time}\times r_{\mathrm{SD}}$ interaction (Fig. 3). These results suggest a common signal contribution, which was likely the scalp considering the limited brain sensitivity for the detector at $r_{\mathrm{SD}}=1\;\mathrm{cm}$ [Fig. 2(b)]. Similar decreases in blood flow measured by DCS at a single source-detector distance have also been reported in response to thigh cuff deflation, which causes a comparable MAP reduction to the rapid-onset LBNP used in the current study.^13^^,^^14^ Further evidence of a substantial signal contribution from the scalp was the large reduction in the DCS BFi measured at both distances when the head tourniquet was inflated (∼80%). These results indicate that the DCS data acquired at $r_{\mathrm{SD}}=2.5\;\mathrm{cm}$ are susceptible to extracerebral signal contamination and should not be treated solely as a marker of CBF.

To isolate the CBF response to LBNP, the multidistance DCS data were analyzed using a two-layer model that incorporated a pressure modulation procedure aimed at assessing the sensitivity of the two distances to scalp blood flow.^35^ The large contrast between the reconstructed CBF time course obtained from the model (Fig. 7) and the blood flow changes measured at the two distances individually (Fig. 3) demonstrates the importance of using multi-layered models to separate scalp and brain signal contributions. Similar approaches have been used to isolate CBF responses to thigh cuff-induced transient hypotension^14^ and to hypercapnia.^31^ To confirm the CBF time course derived from the model, the LBNP experiment was repeated after inflating a pneumatic tourniquet to impede scalp blood flow. In agreement with the modeling results, the measured blood flow response showed no significant decrease during LBNP (Fig. 7). Similarly, no significant reduction in MCAv as measured by TCD was found (Fig. 7).

The small discrepancy between the average reductions in MCAv and DCS-CBF could be explained by the error in the DCS analysis introduced by assuming an average scalp/skull thickness of 1.2 mm. To investigate the impact of this assumption, the analysis of the LBNP data was repeated while varying the distance to the brain from 1 to 1.4 cm. The corresponding CBF reductions ranged from −1.10%±2.01% to −2.08%±3.91%, none of which were significantly different from baseline (all p≥0.13), indicating that the cerebral microvasculature had minimal response to LBNP. The discrepancy between MCAv and DCS, despite neither being significant, may have been due to increased vascular compliance, designed to protect the microcirculation from hypoperfusion.

The considerable differences in blood flow response in brain and scalp to LBNP illustrate the expected differences in flow regulation in central and peripheral tissues. Myogenic, metabolic, endothelial, and neurogenic mechanisms are involved in the maintenance of CBF during fluctuations in blood pressure. However, the degree of flow regulation is not as critical to scalp tissue considering the low metabolic demands of resting skin and muscle and its ability to store oxygen in myoglobin. The contrasting responses between scalp and brain have also been observed with respect to vascular reactivity to hypercapnia. While it is well known that the cerebral vasculature is exquisitely sensitive to changes in arterial ${\mathrm{CO}}_{2}$ tension,^49^ vascular reactivity in the scalp is considerably muted and slower by comparison.^3^ Considering that blood pressure management is a central component of critical care, differences in vascular regulation between brain and scalp again highlight the need for multi-layer modeling approaches for disentangling flow changes in the two tissues.

The lack of cerebral response to the rapid-onset LNBP was also observed in the trNIRS results. Although multidistance measurements can be combined with trNIRS to improve the separation of the scalp and cerebral components, a time gate positioned at the rising edge of a DTOF recorded at a distance typically used for neuromonitoring ($r_{\mathrm{SD}}=3\;\mathrm{cm}$) will be predominately sensitive to extracerebral tissue.^32^ Likewise, a time gate placed at the trailing edge of a DTOF or a higher moment will provide greater sensitivity to the brain. Using the depth-sensitivity built into time-of-flight data, this study demonstrated that oxygenation changes sensitive to superficial tissue were significantly reduced during LBNP (Fig. 4). Reduced ${\mathrm{StO}}_{2}$ during LBNP has been reported in previous studies using commercial NIRS systems.^50^^,^^51^ However, scalp contamination could not be ruled out as ${\mathrm{StO}}_{2}$ also correlated with skin blood flow measured by laser Doppler flowmetry during LBNP.^52^ In the current study, none of the oxygenation metrics derived from either the late gate or variance exhibited a significant change in response to LBNP (Fig. 5). Furthermore, ${\mathrm{StO}}_{2}$ changes obtained from these two depth-sensitive methods were significantly smaller than the changes derived from the early gate and ΔN analyses, reflecting the differences in scalp and brain vascular responses to a sudden drop in blood pressure. The total hemoglobin concentration was used as a marker of relative blood volume changes, and the negligible decrease during LBNP (Fig. 5) confirms the CBF results obtained from the multidistance DCS data.

A limitation of deriving CBF from a multi-layer model is the increase in the number of variables, including the optical properties of the different tissue layers and the thicknesses of the extracerebral layers.^23^ While trNIRS provided estimates of baseline optical properties in the current study, estimates of scalp and skull thickness were based on previously MRI measurements. As discussed above, calculating the rCBF response to LBNP using a range of skull/scalp thicknesses did not substantially change the time course shown in Fig. 7. However, the uncertainty introduced using an assumed value likely contributed to high inter-subject variability observed in $F_{S,0}$ and $F_{C,0}$ (Table 1).^53^ In comparison, inter-subject variability in baseline CBF is typically <20%.^36^ It is noteworthy that the two participants with the largest discrepancies in baseline $\mu_{a,0}$ (numbers 3 and 8) and highest baseline $\mu_{s0}^{\prime }$ values ($10.4\;{\mathrm{cm}}^{-1}$ and $10.9\;{\mathrm{cm}}^{-1}$, respectively) contributed the most to the intersubject variability in $F_{C,0}$. Removing these two lowered the variability from ∼40% to 23%. Note that the outcomes of the LBNP experiment were the same with and without including these participants. Incorporating the extracerebral thickness as an additional fitting parameter in the optimization routine has been suggested and warrants further investigation.^35^^,^^54^

## 5 Conclusions

The current study demonstrated significantly greater changes in extracerebral hemodynamics than cerebral hemodynamics during a period of transient hypotension. These findings illustrate the importance of accounting for extracerebral signal contamination with DCS and NIRS measures of cerebral hemodynamics during standard physiological paradigms for evaluating cerebral autoregulation. Specifically, we demonstrated that multilayered analysis applied to DCS data acquired at two source-detector distances and trNIRS substantially reduced the effects of extracerebral signal contamination on cerebral hemodynamic and oxygenation measurements during transient hypotension. The lack of a substantial cerebral hemodynamic response was further confirmed by simultaneously measuring MCAv by TCD and by repeating the LBNP challenge while scalp blood flow was impeded by a head tourniquet, which resulted in a substantially lower blood flow response measured by DCS.

## Code, Data, and Materials Availability

The datasets generated during and/or analyzed during the current study are available from the corresponding author on reasonable request.

## References

[1] Ayaz H. et al., “Optical imaging and spectroscopy for the study of the human brain: status report,” Neurophotonics 9(S2), S24001 (2022). 10.1117/1.NPh.9.S2.S24001 36052058 PMC9424749

[2] Durduran T. Yodh A. G., “Diffuse correlation spectroscopy for non-invasive, micro-vascular cerebral blood flow measurement,” Neuroimage 85, 51–63 (2014). NEIMEF 1053-8119 10.1016/j.neuroimage.2013.06.017 23770408 PMC3991554

[3] Milej D. et al., “Characterizing dynamic cerebral vascular reactivity using a hybrid system combining time-resolved near-infrared and diffuse correlation spectroscopy,” Biomed. Opt. Express 11(8), 4571 (2020). BOEICL 2156-7085 10.1364/BOE.392113 32923065 PMC7449704

[r4] Rajaram A. et al., “Optical monitoring of cerebral perfusion and metabolism in adults during cardiac surgery with cardiopulmonary bypass,” Biomed. Opt. Express 11(10), 5967 (2020). BOEICL 2156-7085 10.1364/BOE.404101 33149999 PMC7587277

[r5] Milej D. et al., “Assessing the relationship between the cerebral metabolic rate of oxygen and the oxidation state of cytochrome-c-oxidase,” Neurophotonics 9(3), 035001 (2022). 10.1117/1.NPh.9.3.035001 35874144 PMC9298853

[r6] Mesquita R. C. et al., “Direct measurement of tissue blood flow and metabolism with diffuse optics,” Philos. Trans. A. Math. Phys. Eng. Sci. 369(1955), 4390–4406 (2011). 10.1098/rsta.2011.0232 22006897 PMC3263785

[7] Buckley E. M. et al., “Diffuse correlation spectroscopy for measurement of cerebral blood flow: future prospects,” Neurophotonics 1(1), 011009 (2014). 10.1117/1.NPh.1.1.011009 25593978 PMC4292799

[8] Rajaram A. et al., “Perfusion and metabolic neuromonitoring during ventricular taps in infants with post-hemorrhagic ventricular dilatation,” Brain Sci. 10(7), 452 (2020). 10.3390/brainsci10070452 32679665 PMC7407524

[r9] Giovannella M. et al., “BabyLux device: a diffuse optical system integrating diffuse correlation spectroscopy and time-resolved near- infrared spectroscopy for the neuromonitoring of the premature newborn brain,” Neurophotonics 6(2), 025007 (2019). 10.1117/1.NPh.6.2.025007 31093515 PMC6509945

[r10] Baker W. B. et al., “Continuous non-invasive optical monitoring of cerebral blood flow and oxidative metabolism after acute brain injury,” J. Cereb. Blood Flow Metab. 39(8), 1469–1485 (2019). 10.1177/0271678X19846657 31088234 PMC6681541

[11] Lee S. Y. et al., “Quantifying the cerebral hemometabolic response to blood transfusion in pediatric sickle cell disease with diffuse optical spectroscopies,” Front. Neurol. 13, 869117 (2022). 10.3389/fneur.2022.869117 35847200 PMC9283827

[12] Kim M. N. et al., “Continuous optical monitoring of cerebral hemodynamics during head-of-bed manipulation in brain-injured adults,” Neurocrit. Care 20(3), 443–453 (2014). 10.1007/s12028-013-9849-7 23653267 PMC3883971

[13] Pham T. et al., “Quantitative measurements of cerebral blood flow with near-infrared spectroscopy,” Biomed. Opt. Express 10(4), 2117 (2019). BOEICL 2156-7085 10.1364/BOE.10.002117 31061774 PMC6484993

[14] Parthasarathy A. B. et al., “Dynamic autoregulation of cerebral blood flow measured non-invasively with fast diffuse correlation spectroscopy,” J. Cereb. Blood Flow Metab. 38(2), 230–240 (2018). 10.1177/0271678X17747833 29231781 PMC5951022

[15] Durduran T. et al., “Diffuse optical measurement of blood flow, blood oxygenation, and metabolism in a human brain during sensorimotor cortex activation,” Opt. Lett. 29(15), 1766 (2004). OPLEDP 0146-9592 10.1364/OL.29.001766 15352363

[16] Aaslid R. Markwalder T. M. Nornes H., “Noninvasive transcranial Doppler ultrasound recording of flow velocity in basal cerebral arteries,” J. Neurosurg. 57, 769–774 (1982). JONSAC 0022-3085 10.3171/jns.1982.57.6.0769 7143059

[17] Sawosz P. et al., “Human skull translucency: post mortem studies,” Biomed. Opt. Express 7(12), 5010 (2016). BOEICL 2156-7085 10.1364/BOE.7.005010 28018721 PMC5175548

[r18] Contini D. et al., “Effects of time-gated detection in diffuse optical imaging at short source-detector separation,” J. Phys. D. Appl. Phys. 48(4), 045401 (2015). JPAPBE 0022-3727 10.1088/0022-3727/48/4/045401

[19] Wu M. M. et al., “Complete head cerebral sensitivity mapping for diffuse correlation spectroscopy using subject-specific magnetic resonance imaging models,” Biomed. Opt. Express 13(3), 1131–1151 (2022). BOEICL 2156-7085 10.1364/BOE.449046 35414976 PMC8973189

[20] Scholkmann F. et al., “A review on continuous wave functional near-infrared spectroscopy and imaging instrumentation and methodology,” Neuroimage 85, 6–27 (2014). NEIMEF 1053-8119 10.1016/j.neuroimage.2013.05.004 23684868

[21] Sato T. et al., “Reduction of global interference of scalp-hemodynamics in functional near-infrared spectroscopy using short distance probes,” Neuroimage 141, 120–132 (2016). NEIMEF 1053-8119 10.1016/j.neuroimage.2016.06.054 27374729

[r22] Kovacsova Z. et al., “Absolute quantification of cerebral tissue oxygen saturation with multidistance broadband NIRS in newborn brain,” Biomed. Opt. Express 12(2), 907 (2021). BOEICL 2156-7085 10.1364/BOE.412088 33680549 PMC7901317

[23] Verdecchia K. et al., “Assessment of a multi-layered diffuse correlation spectroscopy method for monitoring cerebral blood flow in adults,” Biomed. Opt. Express 7(9), 3659 (2016). BOEICL 2156-7085 10.1364/BOE.7.003659 27699127 PMC5030039

[24] Kacprzak M. et al., “Time-resolved imaging of fluorescent inclusions in optically turbid medium - phantom study,” Opto-Electron. Rev. 18(1), 37–47 (2009). OELREM 1230-3402 10.2478/s11772-009-0027-6

[r25] Blaney G. et al., “Multi-distance frequency-domain optical measurements of coherent cerebral hemodynamics,” Photonics 6(3), 83 (2019). 10.3390/photonics6030083 34079837 PMC8168742

[r26] Liebert A. et al., “Bed-side assessment of cerebral perfusion in stroke patients based on optical monitoring of a dye bolus by time-resolved diffuse reflectance,” Neuroimage 24(2), 426–435 (2005). NEIMEF 1053-8119 10.1016/j.neuroimage.2004.08.046 15627584

[27] Carp S. A. et al., “Combined multi-distance frequency domain and diffuse correlation spectroscopy system with simultaneous data acquisition and real-time analysis,” Biomed. Opt. Express 8, 3993–4006 (2017). BOEICL 2156-7085 10.1364/BOE.8.003993 29026684 PMC5611918

[28] Sutin J. et al., “Time-domain diffuse correlation spectroscopy,” Optica 3(9), 1006 (2016). 10.1364/OPTICA.3.001006 28008417 PMC5166986

[29] Carp S. A. Robinson M. B. Franceschini M. A., “Diffuse correlation spectroscopy: current status and future outlook,” Neurophotonics 10(1), 013509 (2023). 10.1117/1.NPh.10.1.013509 36704720 PMC9871606

[30] Selb J. et al., “Sensitivity of near-infrared spectroscopy and diffuse correlation spectroscopy to brain hemodynamics: simulations and experimental findings during hypercapnia,” Neurophotonics 1(1), 015005 (2014). 10.1117/1.NPh.1.1.015005 25453036 PMC4247161

[31] Milej D. et al., “Direct assessment of extracerebral signal contamination on optical measurements of cerebral blood flow, oxygenation, and metabolism,” Neurophotonics 7(4), 045002 (2020). 10.1117/1.NPh.7.4.045002 33062801 PMC7540337

[32] Milej D. et al., “Incorporating early and late-arriving photons to improve the reconstruction of cerebral hemodynamic responses acquired by time-resolved near-infrared spectroscopy,” J. Biomed. Opt. 26(5), 056003 (2021). JBOPFO 1083-3668 10.1117/1.JBO.26.5.056003

[33] Claassen J. A. H. R. et al., “Regulation of cerebral blood flow in humans: physiology and clinical implications of autoregulation,” Physiol. Rev. 101(4), 1487–1559 (2021). PHREA7 0031-9333 10.1152/physrev.00022.2020 33769101 PMC8576366

[34] Goswami N. et al., “Lower body negative pressure: physiological effects, applications, and implementation,” Physiol. Rev. 99(1), 807–851 (2019). PHREA7 0031-9333 10.1152/physrev.00006.2018 30540225

[35] Baker W. B. et al., “Pressure modulation algorithm to separate cerebral hemodynamic signals from extracerebral artifacts,” Neurophotonics 2(3), 035004 (2015). 10.1117/1.NPh.2.3.035004 26301255 PMC4524732

[36] Milej D. et al., “Quantification of cerebral blood flow in adults by contrast-enhanced near-infrared spectroscopy: validation against MRI,” J. Cereb. Blood Flow Metab. 40(8), 1672–1684 (2020). 10.1177/0271678X19872564 31500522 PMC7370369

[37] Khalid M. et al., “Development of a stand-alone DCS system for monitoring absolute cerebral blood flow,” Biomed. Opt. Express 10(9), 4607 (2019). BOEICL 2156-7085 10.1364/BOE.10.004607 31565512 PMC6757462

[38] Kewin M. et al., “Evaluation of hyperspectral NIRS for quantitative measurements of tissue oxygen saturation by comparison to time-resolved NIRS,” Biomed. Opt. Express 10(9), 4789 (2019). BOEICL 2156-7085 10.1364/BOE.10.004789 31565525 PMC6757477

[39] Abdalmalak A. et al., “Using fMRI to investigate the potential cause of inverse oxygenation reported in fNIRS studies of motor imagery,” Neurosci. Lett. 714, 134607 (2020). NELED5 0304-3940 10.1016/j.neulet.2019.134607 31693928

[40] Liu H. et al., “Influence of blood vessels on the measurement of hemoglobin oxygenation as determined by time-resolved reflectance spectroscopy,” Med. Phys. 22(8), 1209–1217 (1995). MPHYA6 0094-2405 10.1118/1.597520 7476706

[41] Liebert A. et al., “Evaluation of optical properties of highly scattering media by moments of distributions of times of flight of photons,” Appl. Opt. 42(28), 5785 (2003). APOPAI 0003-6935 10.1364/AO.42.005785 14528944

[42] Milej D. et al., “Optimization of the method for assessment of brain perfusion in humans using contrast-enhanced reflectometry: multidistance time-resolved measurements,” J. Biomed. Opt. 20(10), 106013 (2015). JBOPFO 1083-3668 10.1117/1.JBO.20.10.106013 26509415

[43] Milej D. et al., “Time-resolved subtraction method for measuring optical properties of turbid media,” Appl. Opt. 55(7), 1507 (2016). APOPAI 0003-6935 10.1364/AO.55.001507 26974605

[44] Abdalmalak A. et al., “Assessing the feasibility of time-resolved fNIRS to detect brain activity during motor imagery,” Proc. SPIE 9690, 969002 (2016). PSISDG 0277-786X 10.1117/12.2209587

[45] Re R. et al., “Multi-channel medical device for time domain functional near infrared spectroscopy based on wavelength space multiplexing,” Biomed. Opt. Express 4(10), 2231–2246 (2013). BOEICL 2156-7085 10.1364/BOE.4.002231 24156079 PMC3799681

[46] Verdecchia K. et al., “Assessment of the best flow model to characterize diffuse correlation spectroscopy data acquired directly on the brain,” Biomed. Opt. Express 6(11), 4288 (2015). BOEICL 2156-7085 10.1364/BOE.6.004288 26600995 PMC4646539

[47] Li G. Bronk J. T. Kelly P. J., “Canine bone blood flow estimated with microspheres,” J. Orthop. Res. 53, 7–11 (1989). JOREDR 0736-0266 10.1002/jor.1100070109 2908913

[48] Strangman G. E. Li Z. Zhang Q., “Depth sensitivity and source-detector separations for near infrared spectroscopy based on the Colin27 brain template,” PLoS One 8(8), e66319 (2013). POLNCL 1932-6203 10.1371/journal.pone.0066319 23936292 PMC3731322

[49] Duffin J. Mikulis D. J. Fisher J. A., “Control of cerebral blood flow by blood gases,” Front. Physiol. 12, 640075 (2021). FROPBK 0301-536X 10.3389/fphys.2021.640075 33679453 PMC7930328

[50] Kay V. L. Rickards C. A., “The role of cerebral oxygenation and regional cerebral blood flow on tolerance to central hypovolemia,” Am. J. Physiol. - Regul. Integr. Comp. Physiol. 310, R375–R383 (2016). 10.1152/ajpregu.00367.2015 26676249

[51] Houtman S. et al., “Changes in cerebral oxygenation and blood flow during LBNP in spinal cord-injured individuals,” J. Appl. Physiol. 91, 2199–2204 (2001). 10.1152/jappl.2001.91.5.2199 11641362

[52] Hisdal J. et al., “Associations between changes in precerebral blood flow and cerebral oximetry in the lower body negative pressure model of hypovolemia in healthy volunteers,” PLoS One 14(6), e0219154 (2019). POLNCL 1932-6203 10.1371/journal.pone.0219154 31251778 PMC6599124

[53] Zhao H. Buckley E. M., “Influence of source–detector separation on diffuse correlation spectroscopy measurements of cerebral blood flow with a multilayered analytical model,” Neurophotonics 9(3), 035002 (2022). 10.1117/1.NPh.9.3.035002 35874143 PMC9299346

[54] Wu J. et al., “Two-layer analytical model for estimation of layer thickness and flow using Diffuse Correlation Spectroscopy,” PLoS One 17(9), 1–20 (2022). POLNCL 1932-6203 10.1371/journal.pone.0274258 PMC9481000 36112634

